# Relationship between the Prevalence of Metabolic Disease and Impaired Mobility, Diet, Physical Activity, and Socio-Demographic Characteristics in the Polish Elderly—A Cross-Sectional Study

**DOI:** 10.3390/life13040864

**Published:** 2023-03-23

**Authors:** Marzena Jeżewska-Zychowicz, Robert Gajda

**Affiliations:** 1 Department of Food Market and Consumer Research, Institute of Human Nutrition Sciences, Warsaw University of Life Sciences (SGGW-WULS), Nowoursynowska 159C, 02-776 Warsaw, Poland; 2 Department of Human Nutrition, Faculty of Biotechnology and Food Science, Wrocław University of Environmental and Life Sciences, Chełmońskiego 37, 51-630 Wroclaw, Poland

**Keywords:** elderly people, metabolic disease, mobility impairment, diet, physical activity

## Abstract

Maintaining good health for as long as possible presents a great challenge for the elderly. As the elderly population is growing, there is an ongoing need to identify health risk factors affecting older individuals. The study aimed to explore relationships between sociodemographic characteristics, diet, physical activity, and prevalence of metabolic diseases and impaired mobility in the Polish elderly. A cross-sectional study was carried out on 417 elderlies in May–July 2021. Cluster analysis was applied to separate four homogeneous clusters based on the prevalence of metabolic disease and impaired mobility. Logistic regression analysis was used to verify associations between variables. Being overweight or having obesity, as well as following a diet, increased the probability of being affected by metabolic disease. Being well educated, having a better financial situation, positive perception of own health, and having at least moderate physical activity decreased the probability of suffering from mobility impairments. Eating behaviors were not found to be predictors of the disease. However, they differentiated the selected clusters. The results confirmed the heterogeneity of factors that may impact healthy aging. Thus, they should be taken into account by public health authorities to develop health promotion actions adjusted to the needs of specific subgroups.

## 1. Introduction

Modern societies are aging due to increasing life expectancy and falling fertility rates [1,2]. Longer life expectancy results in an increasing number of older people who are at risk of disease and disability [3], determined by several common factors. One of these is poor diet and associated conditions [4,5], such as being overweight and having obesity [6], metabolic syndrome [7], or malnutrition [8,9]. On the other hand, illness can also become a factor that affects the diet. The need to change existing dietary patterns because of an illness can lead to negative consequences (such as eliminating some food from the diet), consequently increasing nutritional risk in the elderly [10]. Negative consequences can also result from limitations in terms of physical and economic accessibility of food, which reduces the food security of the elderly. A significant number of elderly households are affected by the risk of food insecurity [11,12], the consequence of which may lead to many harmful health effects [13].

In addition to an unhealthy diet, physical inactivity is considered a key risk factor for the global burden of disease [14,15]. With the increased energy expenditure, physically active older adults experience healthier aging trajectories, a better quality of life, and better cognitive functioning [16,17].

Poor diet and insufficient physical activity lead to increased metabolic risk factors, such as hypertension, dyslipidemia, impaired glucose metabolism, insulin resistance, or obesity, which can coexist, creating the phenomenon of metabolic multimorbidity [18,19]. Metabolic multimorbidity contributes to the deterioration of the quality of life [20]. However, it is less likely to impair the instrumental activities and mental health of patients compared to other patterns of multimorbidity [21].

Human mobility includes several domains, ranging from physical, cognitive, and neuromuscular to psychological domains [22]. Mobility limitations are highly prevalent with increasing age and have been associated with an increased disability and mortality risk, as well as decreased quality of life and poor psycho-social health [23,24]. Mobility limitations can be linked to both a low activity level [25] and chronic diseases, in particular musculoskeletal, cardiovascular, and neurological diseases [26,27].

Elderly people living in their own homes, such as those living in nursing homes, face health risks for similar reasons, mostly related to age. Nevertheless, these individuals retain the ability to make their own choices and interact with others [28], although various physical and mental limitations can sometimes severely limit their independence [29,30]. The literature on the subject contains many studies on the health status of the elderly, their mental functioning, and physical activity [29,31,32]. However, there is less up-to-date research on factors that contribute to health risk among the elderly living in their own homes [10,33] than among the institutionalized elderly [34,35].

Maintaining good health and well being for as long as possible presents a great challenge for the elderly [36]. For this reason, but also because of the increase in the elderly population, it becomes necessary to assess and identify risk factors in the elderly to improve their health and quality of life [2,37,38]. The consideration of some factors linked to diet and physical activity may be important in preventing a disease-related reduction in quality of life among the elderly.

Therefore, this study aimed to investigate the associations between socio-demographic characteristics, diet, physical activity, and prevalence of metabolic disease and mobility impairment in a group of Polish elderly. We assumed that people with different diseases differ in terms of individual socio-demographic characteristics, but also in terms of their diets and physical activity. Thus, we hypothesized that individuals who suffer from a particular disease are more likely to display some habits causing health risks than healthy individuals. However, the habits, causing health risks, are associated with the type of disease. It seemed more likely that the lifestyle of disease-stricken elderly, including diet and physical activity, will be less healthy compared to their peers not suffering from the disease. At the same time, diet seems to be a factor of greater importance for those with metabolic disease and physical activity than for those with mobility impairment.

## 2. Materials and Methods

### 2.1. Study Design and Sample

A cross-sectional study was carried out in two Polish regions, i.e., the Świętokrzyskie and the Śląskie/Dolnośląskie region. The survey was conducted between May and July 2021 among Polish people aged 60 and over. The survey sample was selected using the snowball method. A total of 900 questionnaires were distributed to 21 clubs, foundations, or other senior citizen organizations in both regions. The recruitment criteria were age 60 and older and residence in the local community. Those who agreed to participate in the study were asked to pass the questionnaire to people in their place of residence who met the age criterion. A total of 466 questionnaires were collected, 49 of which were rejected due to missing responses. The survey sample consisted of 417 participants. The study was conducted by the Declaration of Helsinki [39]. Informed consent was obtained from all participants to participate in the study.

### 2.2. Questionnaire

The incidence of two health problems in the study group was assessed using the following questions: “Do you suffer from any metabolic disease diagnosed by a doctor (e.g., diabetes, hypertension, atherosclerosis, obesity) (answer Yes/No) and “Do you suffer from any disease that impairs your mobility?” (answer Yes/No).

Selected questions from the seniors in the community: Risk Evaluation for Eating and Nutrition (SCREEN-14) questionnaire were used to assess nutritional factors [40]. The questions addressed the previous six months in terms of occurrence of situations, such as skipping meals (answers: never, sometimes, often, almost every day), the number of servings of vegetables and fruits consumed per day, the frequency of consumption of such products as meat, fish, eggs and legumes, and the frequency of consumption of milk and dairy products (answers: less than once a day, usually once a day, one to two times a day, two or more times a day), the number of drinks consumed (answers: two glasses or less, three to four glasses, five to seven glasses, or eight glasses or more), as well as the occurrence of difficulty chewing and biting and swallowing (answers: never, rarely, sometimes, often or always).

The following questions were used to characterize the study group:

1. Socio-demographic characteristics: gender (female/male), age (in years), education (primary, vocational, high school, higher), place of residence (rural/urban area), occupational status (worker/retirement or pension),
2. Financial situation:
  I live very poorly—I do not have enough even for basic needs; I live very modestly—I have to be very frugal daily—financial situation “below average”;
  I live on an average level—there is enough for a daily basis, but I have to save for more serious purchases—“average” financial situation;
  I live well—I have enough for a lot without special savings; I live very well—I can afford some luxury—financial situation “above average”,
3. Health characteristics: subjective assessment of one’s own health (worse than peers, same, better than peers), height, and weight, from which BMI was calculated. Participants’ BMI categories were assigned according to the World Health Organization: normal weight (18.50 ≤ BMI ≤ 24.99 kg/m^2^), overweight (25.00 ≤ BMI≤ 29.99 kg/m^2^), and obesity (BMI ≥ 30.00 kg/m^2^).
4. Characteristics of the family situation: family status (I live alone; I live with my husband/wife; I live with my family, but without my husband/wife; I live with my husband/wife and with my family) and assessment of family relations (very good, good, average, bad, very bad).
5. Lifestyle: dieting (Yes/No); frequency of drinking alcohol (never, one to three times a month or more rarely; once a week or more often); physical activity in leisure time/outside leisure time (small, moderate, high). The description of the scale was different for leisure and work/home time. ‘Low’ physical activity in leisure time was described as a ‘sedentary lifestyle, watching TV, reading the press, books, light housework, taking a walk for 1–2 h a week’; ‘moderate’ one as ‘walks, cycling, gymnastics, gardening or other light physical activity performed for 2–3 h a week’, and ‘high’ one as ‘cycling, running, working on a plot or garden, and other sports activities requiring physical effort, taking up more than 3 h a week’. The description of activity at work/home time was as follows: ‘low‘—‘over 70% of the time in a sitting position’; ‘moderate’—‘approximately 50% of the time in a sitting position and moving for about 50% of the time’; and ‘high’—‘being in motion for about 70% of the time or doing physical work associated with a lot of effort’ [Beliefs …, 2014].

### 2.3. Statistical Analysis

Descriptive statistics were used to present the characteristics of the study sample. A K-means cluster analysis was applied using two variables, namely, declaration of suffering from a metabolic disease or problems with mobility to separate homogeneous groups (clusters). The profiling of the clusters was carried out with the use of sociodemographic variables, variables describing eating habits, and self-reported physical activity. The Chi-square test was used to assess the diversity between groups. A *p*-value lower than 0.05 was considered significant.

Logistic regression analysis was used to verify associations between sociodemographic variables, variables describing eating habits and lifestyle (independent variables), and the prevalence of metabolic disease, as well as diseases causing mobility impairment (dependent variables). Odds ratios (ORs) represented the probability of adherence to the group with the disease. The reference groups (OR = 1.00) were those that declared neither having a metabolic disease nor disease-causing mobility problems. Wald’s test was used to assess the significance of ORs.

All analyses were performed with IBM Statistics SPSS, version 27.0 (IBM Corp., Armonk, NY, USA).

## 3. Results

### 3.1. Characteristics of the Study Sample

Table 1 displays the characteristics of the study sample. The majority of respondents were women (74.8%), while men were significantly less represented (25.2%). More respondents (70.7%) lived in urban areas compared to rural areas (29.3%). More than 1/3 of the respondents (36.9%) lived alone. The majority (59.7%) rated their health as the same as their peers. Only 27.1% of respondents were of normal weight, while the rest were overweight or obese. More than half of the study sample (52.3%) had a metabolic disease, and 21.1% had a disease that impaired their mobility. The average age was 70.8 years (SD = 6.73).

### 3.2. Socio-Demographic and Health Characteristics of the Clusters

The clusters identified according to the presence of metabolic or mobility-impairing disease are presented in Table 2.

The clusters differed by age, family status, self-assessed economic situation, and employment status (Table 3). The metabolic disease cluster was dominated by people between 65 and 70 years old (41.2%), and the fewest people between 60 and 65 years old (15.0%). In addition, it had more people (64.1%) with an average financial situation (enough for daily living, but I need to save for more serious purchases) than the other clusters. In the cluster of people declaring the absence of metabolic disease and mobility impairment, there were more people aged 60–65 (30.1%), people living alone (39.8%), people with above-average financial status (38.6%), and professionally active people (24.4%) compared to the other clusters.

The cluster of respondents declaring the presence of both a metabolic disease and a mobility impairment had the highest number of people between the ages of 71 and 75 (30.8%), those living alone—38.5% (percentage similar to the group of people without both diseases), or living with family (36.9%) compared to the other clusters. In contrast, the cluster of people declaring the presence of a mobility impairment was characterized by the highest number of people who are retired or on a pension (95.7%), people over 75 years old (56.5%), living with a partner (56.5%), and those with a below-average financial status (34.8%) than in the other clusters (Table 3).

Family relations described as worse than good were indicated by more than 1/3 of respondents with a mobility impairment (34.8%) and about 1/4 of those with both diseases (23.1%). Fewest people with metabolic disease expressed such opinions (9.8%), while most of these respondents (49.0%) described family relations as very good (Table 3).

Approximately half of the seniors with both diseases or with only a mobility-impairing disease (53.8% and 47.8%, respectively) rated their health as worse than their peers. More than one-fifth of those with a metabolic disease or without both diseases (22.9% and 23.3%, respectively) rated their health as better than their peers (Table 3). The cluster with mobility impairment had the most people with obesity (34.8%) compared to the other clusters. In contrast, the group without both diseases included more people with normal weight (36.4%) and the smallest percentage of respondents with obesity (15.3%). The difficulties in swallowing (36.9%) and biting and chewing (35.4%) were reported most often by people with both diseases, and the least often with regard to such difficulties (11.8% and 13.1%, respectively) were reported by people with a metabolic disease, and in the case of swallowing difficulties, this was also reported by people with a mobility impairment (8.7%) (Table 3).

### 3.3. Cluster Characteristics including Diet and Physical Activity

Characteristics of the identified clusters in terms of diet and physical activity are displayed in Table 4. Following a specific diet was characteristic for respondents with metabolic disease (30.7%) and with both diseases (35.4%), and the fewest people followed a diet among those with mobility impairment (4.3%). Frequent skipping of meals characterized the largest percentage of people with both diseases (26.2%), while the largest number of people with a mobility impairment never or very rarely skipped meals (52.2%). Skipping meals from time to time was declared by the largest percentage of people without both diseases (51.1%). The smallest number of people with a metabolic disease declared frequent consumption of milk and dairy products (two to three times a day or more often) (7.8%). In the group of people with mobility problems, this group of products was consumed two to three times a day or more frequently by 30.4% of respondents, while the same number of seniors consumed them less than once a day (30.4%). People with mobility problems drank the most (26.1% reported drinking eight glasses of drinks or more per day), but this cluster also had the highest number of people drinking two glasses or less (13.0%). The frequency of consumption of meat, fish, eggs, and legumes, but also servings of vegetables and fruits, did not vary in clusters. 

The results of logistic regressions are presented in Table 5. They have demonstrated that people who declared suffering from a metabolic disease were more likely to be overweight (OR: 1.93, 95% CI 1.16; 3.22) and obese (OR: 3.13, 95% CI 1.67; 5.88). They were less likely to perceive their health as the same as their peers (OR: 0.32, 95% CI 0.16; 0.63) or better than their peers (OR: 0.37, 95% CI 0.17; 0.82).

Respondents with a mobility impairment were less likely to have high school education (OR: 0.30, 95% CI 0.12; 0.77) and higher education (OR: 0.27, 95% CI 0.09; 0.81). They were also less likely to declare an average financial situation (OR: 0.30, 95% CI 0.12; 0.77) and an above-average financial situation (OR: 0.28, 95% CI 0.09; 0.81). Moreover, they were less likely to perceive their health as the same (OR: 0.10, 95% CI 0.04; 0.22) or better than peers (OR: 0.07, 95% CI 00.02; 0.22).

People who declared suffering from metabolic disease or mobility problems were more likely to be on a diet (OR: 3.01 95% CI 1.18; 5.11; OR: 2.38, 95% CI 1.18; 4.76, respectively). People with mobility problems were less likely to declare moderate and high physical activity (OR: 0.39 95% CI 0.17; 0.91; OR: 0.30, 95% CI 0.10; 0.91, respectively) and to drink alcohol once a week or more often (OR: 0.22 95% CI 0.09; 0.79) (Table 5).

## 4. Discussion

The aim of this study was to explore the links between the prevalence of metabolic and mobility-impairing diseases in a group of Polish elderly and their socio-demographic characteristics, as well as some lifestyle factors. The identification of four homogeneous clusters based on declared diseases and then profiling them due to selected variables provided insight into these relationships. The socio-demographic characteristics that distinguished the clusters from each other were age, family status, but also financial and employment status. In addition, differences were found after taking into account self-reported family relations and health status, BMI, and the presence of difficulties in swallowing, biting, and chewing. Among lifestyle attributes, differences between clusters were found in both eating habits and physical activity. Thus, the assumption of differences regarding individual characteristics and lifestyles between elderly people with various diseases and, furthermore, between them and healthy people were confirmed.

The absence of both metabolic diseases and mobility impairments was characteristic of people aged 60–65, living alone, professionally active, and with above-average financial status. Most people with metabolic diseases were between 65 and 70 years old and declared an average financial situation, whereas the largest number of respondents with both metabolic diseases and mobility impairments were between 71 and 75 years old and lived alone or with family. The mobility impairment was declared by more respondents who were retired or on a pension, over 75 years old, lived with a partner, and those with a below-average financial status. The demonstrated differentiation of the group by age is consistent with other studies showing that progressive degeneration of tissues that occurs with age has a negative impact on the structure and function of vital organs, which promotes the successive appearance of diseases [41,42,43]. Although, until age 80, most people do not have functional impairment or disability [44], in our study some respondents aged 71–75 years belonged to the cluster with both metabolic diseases and mobility impairments.

Previous research shows that residential/family status is crucial for the quality of life of the elderly [45,46]. Those who live with their spouse are more capable of coping with poor health [46]. In this study, among respondents living on their own, those with mobility difficulties were the least represented, while the largest number of individuals struggling with such an impairment lived only with their partner. Not being independent might explain the need to live with other people. However, in this group, there were few people living with their families. In addition, the largest number of people with mobility impairment (34.8%) negatively assessed their family relations (“worse than good”). This may result from the fact that those elderly did not receive support from family, especially when they already needed more specialized care or treatment from professional medical institutions [47], and they are not available. Some policies, such as community care, integrated medical care, and other forms of care can encourage the elderly to not live with their children when they need care, as they can obtain necessary life care from public services [47]. However, in Poland, such social assistance is still not widely accessible due to the lack of development of services provided in the vicinity of the place of residence [48]. The deterioration of health conditions may increase the tendency to live only with one’s spouse [49], which in turn may explain the results of the survey, indicating that more than half of people with mobility limitations (56.5%) lived only with their partner. Previous research showed that the elderly with multiple physical disabilities and impaired activities of daily living preferred not to live with their children [47,49]. Nevertheless, further research should be conducted in this area, with a focus both on the availability of social assistance and the family situation.

Our results regarding the financial situation of seniors are consistent with what is well known, namely, that the prevalence of chronic diseases is higher among poor older people [50]. A worse than the average financial situation was declared by more people with both types of diseases, and in addition, the fewest people with mobility difficulties described it as above average. The relationship between chronic diseases and the financial situation is two-sided, as financial constraints can be both a cause and an effect of chronic diseases. Previous studies have shown that older people with diagnosed chronic diseases face huge health expenditures even in some of the wealthiest countries in Europe [51,52,53], but also in Poland [53]. In such a situation, it is crucial to strengthen the mechanisms for the financial protection of older people with chronic diseases to protect them against health expenditures. An underestimation of the extent of the financial struggles of the elderly may also be a limitation, as older adults may feel that their financial resources are adequate even when they are low [50], which can be a barrier to providing them with financial assistance or health care. Thus, the development of effective strategies for the financial protection of the older part of the population requires more information on who is affected and at which point of their illness trajectory they are.

No differences were shown between clusters in terms of gender, which is inconsistent with other studies according to which gender differentiates the incidence of diseases among the elderly [45,54]. While men suffer more than women from cancer and heart disease, which are key factors of mortality, women, in turn, have higher rates of chronic conditions, such as arthritis, depression, osteoporosis, and related fractures. Such diseases cause suffering but threaten life less than cancer and heart disease do [55,56]. This is known as the gender paradox [54]. One of the explanations for the gender paradox in morbidity vs. longevity is based on gender differences in lifestyle, especially habits of smoking and alcohol consumption, which are strongly related to mortality in men. The differences in the types of chronic diseases and other health conditions suffered by men and women, as well as the differential prevalence of behavior-related risk factors, can partly explain the reports regarding gender differences in general life expectancy but also in the life expectancy of individuals with disability [57]. In contrast, there were no differences between clusters after accounting for gender but also for place of residence in the study sample. Research among the Polish elderly confirms the existence of differences between men and women in terms of various health indicators, including the incidence of diseases and hospitalizations [58,59] and lifestyle [60]. Moreover, Polish medical care suffers from gender bias, which possibly makes men more responsive to medical care [61]. Thus, these obtained results should be interpreted cautiously from the perspective of the gender paradox. The inclusion of only elderly people living in their own homes in the study group can be considered to be an explanation for such differences. Further research aimed at confirming differences in health indicators, taking into account residence status is advisable.

In the study sample, there were 73% of people that were overweight and obese people, including 24% of people with obesity, while in the the Polsenior 2 national survey, there were more obese people (38%) and fewer overweight people (39%) [59]. The smallest number of obese people were among those without both diseases, which is consistent with the results of many studies, showing that the prevalence of some diseases (including hypertension, metabolic syndrome, and coronary heart disease) increases with increasing obesity in the elderly [62]. In the study group, the smallest number of respondents with mobility difficulties or metabolic disease had normal weight, while the absence of both diseases was associated with a two-fold higher percentage of respondents with normal weight. These results in part reflect an obesity paradox [63], showing that the elderly have a higher risk of morbidity with increasing BMI values. However, an inverse relationship is observed for mortality [64]. When interpreting the obtained results, it should be remembered that the BMI according to WHO criteria for the elderly is beginning to be questioned because “normal weight” seems less reliable for older adults. The WHO criteria may obscure a problem that exists in terms of determining the health of the older population, namely, that weight loss is actually detrimental to health [65]. Some studies suggest that it is better for older adults to have a BMI of 25–35 kg/m^2^ to maintain their functionality and reduce the risk of falling [66]. In the study group, BMI was one of the predictors of metabolic diseases, namely, people suffering from metabolic diseases were more likely to be overweight or obese in comparison with those with normal weight. Thus, these results confirm that the prevalence of some diseases in the elderly may correlate with increased body weight, especially with obesity [62].

The occurrence of various diseases can promote favorable changes in the diet of the elderly [67], but it can also lead to adverse changes that increase nutritional risk [10]. In the study sample, the frequent skipping of meals was observed in the group of people with both diseases (26.2%). One of the consequences of such a habit may be a decreased food intake. The decrease in food intake may also be influenced by many changes in the body, including difficulties related to food intake, changes in gastrointestinal function, changes in hormonal balance affecting satiety signals, and a reduction in sensory-specific satiety [68,69]. Difficulties in biting, chewing, and swallowing, which affected about one-fifth of the study sample, were observed by more than 35% of the respondents with both diseases. In contrast, the identified clusters did not differ in terms of frequency of consumption of vegetables and fruits, but also protein-rich foods (meat, fish, eggs, legumes). This may suggest that the presence of the disease did not modify these behaviors, and they are conditioned by other factors. However, differences were noted in the frequency of consumption of milk and dairy products. Among respondents with mobility impairments, there were most people consumed these products two or more times a day, while more respondents with the metabolic disease rarely consumed milk and dairy products. This could impair the functioning of the latter, as several studies showed that dairy consumption was inversely associated with the occurrence of one or several facets of the metabolic syndrome [70,71]. Previous research showed that greater adherence to a healthy dietary pattern was associated with a lower risk of metabolic syndrome [72,73], while in the study group, there were no differences in the frequency of fruit and vegetable consumption between clusters. A common problem of elderly people is insufficient hydration of the body [74,75]. In the study group, almost 2/5 of people (38.9%) drank at most four glasses of beverages per day, which is considered risky behavior. Differences between clusters were recorded, as most people (74%) with mobility impairment (cluster 4) drank more than four glasses of drinks per day. According to the obtained results, the assumption that respondents with different diseases differ in their diets was confirmed. 

The demonstrated differences in eating behaviors between clusters can be linked to specific dietary restrictions. Seniors with mobility difficulties were characterized by the smallest number of risky behaviors. At the same time, only 4.3% of these respondents declared that they followed a diet, which is over three times less compared to people without diseases and over seven times less than in the group of people with a metabolic disease or both diseases. The use of a diet increased the chances of developing a metabolic disease threefold. Therefore, it is of great importance to conduct nutritional education activities in a group of elderly people adapted to the needs of this group, especially those suffering from many diseases. This will allow for minimizing the negative consequences of improper use of the diet. The demonstrated differences in eating behaviors of people with both diseases (cluster 3) compared to the group with one of these diseases confirm the legitimacy of such a procedure, as the results of the study indicated differences in terms of skipping meals, the frequency of consumption of milk and dairy products, and the number of fluids consumed.

### Limitations of the Study

The study was cross-sectional, and the cause-and-effect relationship between the variables cannot be fully established. Due to the lack of representativeness of the study group (only two regions were represented), the results of the study cannot be applied to the entire Polish population. The body mass index used in the study to determine nutritional status in the elderly population also has limitations. The use of BMI may lead to an underestimation of the real prevalence of obesity in the senior population. This is due, among other things, to the age-related change in body composition.

## 5. Conclusions

Some individual characteristics of the respondents were found to be predictors of the prevalence of metabolic disease and impaired mobility. Being overweight or obese, as well as following a diet, increased the probability of being affected by metabolic disease. On the other hand, perceiving one’s health as compared to peers’ health decreased the likelihood of the occurrence of such a disease. Being well educated, having a better financial situation, perceiving own health as the same or better compared to peers, exercising at least moderate physical activity, and drinking alcohol at least once a week decreased the probability of suffering from mobility impairment. Similarly to metabolic disease, following a diet increased the chances of respondents being affected by a mobility impairment.

Eating behaviors that are perceived as sources of nutritional risk in old age were not found to be predictors of the disease, but they differentiated the selected clusters. Differences occurred after taking into account skipping meals, frequency of drinking milk, and eating milk products, and the number of consumed beverages, with only people with mobility impairment showing less risky behavior compared to other respondents.

The obtained results confirmed the heterogeneity of factors that may impact healthy aging, including demographic and social-cultural factors (i.e., age, and living alone). Thus, they should be taken into account by public health authorities in order to develop health promotion actions adjusted to the needs of specific population subgroups. However, further research with longitudinal data is needed to improve the understanding of successful aging transitions over a longer life course.

## Figures and Tables

**Table 1:** Characteristics of the study sample.

		Number of Respondents	%
Gender	Women	312	74.8
	Men	105	25.2
Age	60–65 years	94	22.5
	66–70 years	141	33.8
	71–75 years	95	22.8
	above 75 years	87	20.9
Place of residence	Rural area	122	29.3
	Urban area	295	70.7
Family status	Living alone	154	36.9
	Living only with a partner	168	40.3
	Living with family	95	22.8
Education	Vocational and lower	149	35.7
	High school	143	34.3
	Higher	125	30.0
Financial status	Below average	47	11.3
	Average	232	55.6
	Above average	138	33.1
Metabolic disease	No	199	47.7
	Yes	218	52.3
Disease causing mobility problems	No	329	78.9
	Yes	88	21.1
Self-reported health status	Worse than peers	84	20.1
	Same as peers	249	59.7
	Better than peers	84	20.2
BMI	Normal weight	113	27.1
	Overweight	204	48.9
	Obesity	100	24.0

**Table 2:** The identified clusters according to the prevalence of metabolic disease and mobility-impairing disease.

Prevalence	Clusters			
	Metabolic Disease	Without Both Diseases	Both Diseases	Mobility-Impairing Disease
	(1)	(2)	(3)	(4)
Metabolic disease	+	−	+	−
Mobility-impairing disease	−	−	+	+
Number of respondents	153	176	65	23

+ means “present”; − means “absent”.

**Table 3:** Socio-demographic and health characteristics of the identified clusters.

	Total % (N)	Clusters				*p*-Value (Chi^2^)
		Metabolic Disease (1)	Without Both Diseases (2)	Both Diseases (3)	Mobility-Impairing Disease (4)	
Age						
60–65 years	22.5 (94)	15.0	30.1	20.0	21.7	<0.001
66–70 years	33.8 (141)	41.2	33.5	26.2	8.7	
71–75 years	22.8 (95)	26.1	18.2	30.8	13.0	
above 75 years	20.9 (87)	17.6	18.2	23.1	56.5	
Family status						
Living alone	36.9 (154)	34.6	39.8	38.5	26.1	0.026
Living only with a partner	40.3 (168)	44.4	40.3	24.6	56.5	
Living with family	22.8 (95)	20.9	19.9	36.9	17.4	
Financial status						
Below average	11.3 (47)	5.2	6.8	29.2	34.8	<0.001
Average	55.6 (232)	64.1	54.5	41.5	47.8	
Above average	33.1 (138)	30.7	38.6	29.2	17.4	
Employment status						
Retirement or pension	83.0 (346)	88.9	75.6	84.6	95.7	0.004
Professionally active	17.0 (71)	11.1	24.4	15.4	4.3	
Self-reported family relations						
Very good	41.2 (172)	49.0	39.8	35.4	17.4	0.005
Good	43.9 (183)	41.2	46.6	41.5	47.8	
Worse than good	14.9 (62)	9.8	13.6	23.1	34.8	
Self-reported health status						
Worse than peers	20.1 (84)	15.0	8.5	53.8	47.8	<0.001
Same as peers	59.7 (249)	62.1	68.2	38.5	39.1	
Better than peers	20.1 (84)	22.9	23.3	7.7	13.0	
BMI						
Normal weight	27.1 (113)	17.6	36.4	27.7	17.4	<0.001
Overweight	48.9 (204)	52.9	48.3	41.5	47.8	
Obesity	24.0 (100)	29.4	15.3	30.8	34.8	
Swallowing difficulties						
Never	61.2 (255)	69.3	60.8	38.5	73.9	<0.001
Rarely	22.3 (93)	19.0	25.0	24.6	17.4	
Sometimes, often, always	16.5 (69)	11.8	14.2	36.9	8.7	
Difficulty in biting and chewing						
Never	57.1 (238)	66.7	55.7	41.5	47.8	0.006
Rarely	22.8 (95)	20.3	23.9	23.1	30.4	
Sometimes, often, always	20.1 (84)	13.1	20.5	35.4	21.7	

**Table 4:** Characteristics of the identified clusters according to nutrition and physical activity.

	Total % (N)	Clusters				*p*-Value (Chi^2^)
		Metabolic Disease (1)	Without Both Diseases (2)	Both Diseases (3)	Mobility-Impairing Disease (4)	
Skipping meals						
Often or almost every day	13.2 (55)	9.8	11.4	26.2	13.0	0.029
Sometimes	47.2 (197)	47.7	51.1	40.0	34.8	
Never	39.6 (165)	42.5	37.5	33.8	52.2	
Frequency of drinking milk and eating milk products						
Less than once a day	23.0 (96)	24.8	21.0	21.5	30.4	0.039
Usually once a day	33.8 (141)	34.6	37.5	30.8	8.7	
1–2 times a day	29.5 (123)	32.7	27.3	27.7	30.4	
2 or more times a day	13.7 (57)	7.8	14.2	20.0	30.4	
Frequency of eating such products as meat, fish, eggs, and legumes						
Less than once a day	17.0 (71)	13.7	18.2	20.0	21.7	0.611
Usually once a day	31.4 (131)	32.7	31.3	32.3	21.7	
1–2 times a day	37.4 (156)	43.1	34.1	32.3	39.1	
2 or more times a day	14.1 (59)	10.5	16.5	15.4	17.4	
Number of servings of vegetables and fruits consumed per day						
Less than two servings	17.7 (74)	20.3	15.3	18.5	17.4	0.752
Two servings	34.3 (143)	33.3	37.5	30.8	26.1	
Three servings	29.5 (123)	32.0	26.1	30.8	34.8	
Four or more servings	18.5 (77)	14.4	21.0	20.0	21.7	
Amount of drinks consumed (in glasses—250 mL)						
2 or fewer	6.5 (27)	5.2	9.1	0.0	13.0	0.014
3–4	32.4 (135)	35.3	29.5	40.0	13.0	
5–7	46.8 (195)	43.8	46.0	55.4	47.8	
8 or more	14.4 (60)	15.7	15.3	4.6	26.1	
Following a diet						
No	76.7 (320)	69.3	85.2	64.6	95.7	<0.001
Yes	23.3 (97)	30.7	14.8	35.4	4.3	
Frequency of alcohol drinking						
Never	44.1 (184)	43.1	39.2	50.8	69.6	0.046
1–3 times a month or less	45.8 (182)	45.8	44.3	41.5	30.4	
Once a week or more	12.2 (51)	11.1	16.5	7.7	0.0	
Physical activity outside leisure time						
Small	23.0 (96)	20.9	14.8	40.0	52.2	<0.001
Moderate	52.3 (218)	52.3	57.4	44.6	34.8	
High	24.7 (103)	26.8	27.8	15.4	13.0	
Physical activity in leisure time						
Small	38.4 (160)	37.3	29.5	56.9	60.9	<0.001
Moderate	48.9 (204)	47.7	56.8	33.8	39.1	
High	12.7 (53)	15.0	13.6	9.2	0.0	

N—number of respondents.

**Table 5:** Odds ratios for the prevalence of metabolic disease and mobility problems according to sociodemographic, health, and lifestyle characteristics sample.

Variables	Metabolic Disease (Ref. without Disease)		Mobility-Impairing Disease (Ref. without Disease)	
	OR 95% CI *	p **	OR 95% CI	*p*
Education (ref. vocational or lower)				
High school	1.30 (0.75; 2.25)	0.342	0.30 (0.12; 0.77)	0.027
Higher	1.14 (0.63; 2.05)	0.670	0.27 (0.09; 0.81)	0.050
Financial status (ref. below average)				
Average	0.74 (0.32; 1.70)	0.479	0.30 (0.12; 0.77)	0.013
Above average	0.58 (0.24; 1.40)	0.223	0.28 (0.09; 0.81)	0.019
BMI (ref. normal weight)				
Overweight	1.93 (1.16; 3.22)	0.011	1.21 (0.56; 2.63)	0.633
Obesity	3.13 (1.67; 5.88)	<0.001	2.22 (0.90; 5.51)	0.086
Self-reported health status (ref. worse than peers)				
The same as peers	0.32 (0.16; 0.63)	<0.001	0.10 (0.04; 0.22)	<0.001
Better than peers	0.37 (0.17; 0.82)	0.015	0.07 (0.02; 0.22)	<0.001
Following a diet (ref. without dieting)				
Using a diet	3.01 (1.18; 5.11)	<0.001	2.38 (1.18; 4.76)	0.015
Physical activity outside leisure time (ref. small physical activity)				
Moderate	0.63 (0.32; 1.23)	0.177	0.39 (0.17; 0.91)	0.030
High	0.52 (0.23; 1.18)	0.115	0.30 (0.10; 0.91)	0.033
Frequency of drinking alcohol (ref. never)				
1-3 times a month or less	1.00 (0.63; 1.57)	0.995	0.80 (0.44; 1.45)	0.460
Once a week or more	0.57 (0.29; 1.10)	0.095	0.22 (0.09; 0.79)	0.017

* Odds ratios with 95% confidence intervals; ** significance level of the Wald’s test.

## Data Availability

Data presented in this study are available upon request from the corresponding author.
